# Social differences associated with the use of psychotropic drugs among men and women aged 65 to 74 years living in the community: the international mobility in aging study (IMIAS)

**DOI:** 10.1186/s12877-015-0083-3

**Published:** 2015-07-20

**Authors:** Gustave Noufou Nana, Boukaré Doulougou, Fernando Gomez, Alban Ylli, Jack Guralnik, Maria Victoria Zunzunegui

**Affiliations:** Department of Social and Preventive Medicine, School of Public Health, University of Montreal, Montreal, QC Canada; Research group on gerontology and geriatrics, University of Caldas, Manizales, Colombia; National Institute of Public Health, Tirana, Albania; Department of Epidemiology and Public Health, Division of Gerontology, School of Medicine, University of Maryland, Baltimore, MD USA

**Keywords:** Psychotropic drugs, Aging, Social differences, Latin America, Canada, Albania

## Abstract

**Background:**

Elderly persons make greater use of psychotropic drugs, but there are few international studies on social differences in the use of these medications. The aim of this study is to examine social differences in the use of psychotropic drugs among persons aged 65–74 years in the International Mobility in Aging Study (IMIAS).

**Methods:**

The sample consisted of 1,995 participants in the IMIAS 2012 baseline study in Saint-Hyacinthe (Canada), Kingston (Canada), Tirana (Albania), Manizales (Colombia), and Natal (Brazil). During home visits, all medication taken by the participants in the previous 15 days was recorded. We then used the Anatomical Therapeutic Chemical classification system to code psychotropic drugs as anxiolytics, sedatives, hypnotics (ASH); antidepressants (ADP); or analgesics, antiepileptics, or antiparkinsonians (AEP). Prevalence ratios for psychotropic drug use according to sex, education, income, and occupation were estimated by fitting a Poisson regression and controlling for demographic and health covariates.

**Results:**

Psychotropic drug use was higher among Canadian participants than among those living outside Canada. Prevalence of AEP drug use was higher for women than men in the Canadian and Latin American sites. In Tirana, antidepressant drugs were rarely used. Socioeconomic differences varied among sites. In the Canadian cities, low socioeconomic standing was associated with higher frequency of psychotropic drug use. In the Latin American cities, elderly people with high education and income levels showed a higher level of antidepressant drug use, while people with manual occupations had a higher use of AEP drugs. In Tirana, ASH drug use was higher among those with low income.

**Conclusion:**

An inverse association was observed between socioeconomic standing and psychotropic drug use in Canada, while the opposite was true in Latin America. Albania was notable for an absence of antidepressant use and greater use of ASH drugs among low-income groups.

## Background

Psychotropic drug use is widespread among elderly persons treated at outpatient clinics [1]. These drugs are often prescribed for a variety of reasons, such as insomnia, chronic somatic disease, or symptoms of depression. Inappropriate use has been reported: for example, while antidepressants were being taken by 20 % of participants with depressive symptoms, they were also being taken by 5–12 % of those without depressive symptoms [2]. This could be related to the multiple indications of these drugs, ranging from depression to sleep disorders and neuropathic pain [3, 4]. Another example is the addiction [5] that can result from prolonged used of anxiolytics [1, 6–8].

Studies have examined factors associated with psychotropic drug use among elderly persons, but the results are sometimes contradictory. In Quebec, the use of anxiolytics, sedatives, and hypnotics, as well as of antidepressants, has been negatively associated with income and education levels of elderly persons [9, 10]. Studies on elderly persons in the United States and in Northern Europe have found no association between income and education and psychotropic drug use [11–13], while in Sweden psychotropic drugs were more often prescribed by psychiatrists to those with the highest levels of education [14]. Among elderly persons in Israel, use of anxiolytics, sedatives, and hypnotics was negatively associated with level of education, while that of antidepressants increased with level of education [1]. In Australia, women, older adults, and persons with lower education were more likely to be antidepressant users [15]. All of these studies were conducted in elderly populations of high-income countries.

The objective of our study was to estimate the association between socioeconomic factors and psychotropic drug use among men and women aged 65–74 years in five different cities in middle- and high-income countries. Our hypothesis was that there are social differences in psychotropic drug use among men and women aged 65–74 residing in these cities and that these differences are research site independent. We expected psychotropic drugs to be more frequently prescribed among populations with lower education, lower income levels, and less skilled employment, as previously reported in most of the studies cited above.

## Methods

Our data come from the International Mobility in Aging Study’s (IMIAS) baseline data. IMIAS is a longitudinal, multicentre, and multidisciplinary study focusing on five cities: Kingston (Ontario, Canada), Saint-Hyacinthe (Quebec, Canada), Tirana (Albania), Manizales (Colombia), and Natal (Brazil).

The study population consists of persons between the ages of 65 and 74 years residing and living in the community. The sample was stratified by sex, and the aim was to recruit 200 men and 200 women at each site. The overall sample size across the five research sites was 1,995 persons. The data were collected in 2012.

In Tirana, Manizales, and Natal, participants were recruited from primary healthcare community centres. At these sites, a random sample of elderly persons registered at the health centre was selected, and participants were contacted directly by our investigators with an invitation to participate in the study. All neighbourhood health centres in Manizales, five neighbourhood health centres serving middle- and low-income populations in Natal, and two neighbourhood health centres in Tirana serving middle-class populations participated in the study. In Kingston and Saint-Hyacinthe, potential participants, randomly chosen from the list of patients registered at the participating clinics, received a letter from their family doctor inviting them to contact our field coordinator to set up an appointment for a home visit [16]. In Saint-Hyacinthe, all patients came from the largest family medicine group clinic, with which more than 80 % of physicians are affiliated and which covers the whole territory of the city. In Kingston, the two clinics included in the study are large, covering the whole Central Kingston area. In Saint-Hyacinthe the sample was stratified by neighbourhood, while in Kingston this stratification was not possible. These indirect methods of recruitment in the Canadian cities were required because the ethics committees for the Canadian sites (Queens University and the University of Montreal) did not authorize researchers to communicate directly with potential participants to invite them to participate in a study. The response rate was around 95 % in Tirana, Manizales, and Natal, and around 30 % in Kingston and Saint-Hyacinthe, where 95 % of the potential participants who indicated interest by calling our research coordinator actually participated.

Participants who made four or more errors on the Leganes cognitive test (LCT), a screening test for dementia, were excluded from the interview stage [17]. The data were collected at participants’ homes by trained investigators. An interview was conducted, followed by anthropometric and blood pressure measures, as well as tests of mobility and cognitive function, all administered in the appropriate language (English, French, Albanian, Spanish, or Portuguese). The participants were asked whether they had taken any prescribed or over-the-counter medications in the past two weeks. If the answer was positive, the interviewer asked participants to show them all medical drug containers and recorded the drug names.

### Dependent variables

The data on psychotropic drug use were collected by identifying which of the drugs used by participants during the period covered by the study were psychotropic (analgesic, antiepileptic, antiparkinsonian, anxiolytic, sedative, hypnotic, or antidepressant). Identification was based on the Anatomical Therapeutic Chemical (ATC) classification system recommended by the WHO [18]. For analysis purposes, we grouped psychotropic drug use into four categories corresponding to four dependent variables: 1) AEP – analgesic, antiepileptic, and/or antiparkinsonian drugs (coded N02, N03, or N04); 2) ASH – anxiolytic and/or sedative/hypnotic drugs (coded N05B or N05C); 3) ADP – antidepressant drugs (coded N06A); and 4) MSN, representing use of at least one psychotropic drug (any drug coded N).

### Independent variables

Socioeconomic status was measured using education, income, and occupation indicators.

Education level was self-declared, in answer to the question, “What is the highest level of schooling you have completed?” The answers were grouped into two categories: ‘less than secondary school’ (for participants who had little education and had not completed secondary school) and ‘secondary and above’ (for those having completed secondary school or above).

Annual income was determined by asking study subjects, “What is your annual income before taxes?” In Canada, annual income was considered low when it was less than $20,001 (Canadian dollars), medium when it was between $20,001 and $40,000, and high when it was more than $40,000. Less than 20,000$ corresponds approximately to “Old Age Security pension” in Canada for a single individual (17,000$) or a surviving spouse (23,000$) [19]. For Latin America and Albania, low income corresponded to an annual income of $5,000 or less, medium income to an annual income from $5,001 to $10,000, and high income to an annual income greater than $10,000. These cut-off points were based on the value of old age pension in Brazil, which is around $360/month. For analysis, we created two categories: low and medium incomes in one, high income in the other.

Occupation was established by asking the question, “What is the profession or occupation you practiced for the majority of your life?” Occupations were classified according to the International Labour Office’s [20] International Standard Classification of Occupations (ISCO-08), which distinguishes among 10 occupational groups. For our study, these groups were split into two categories: ‘Non-manual occupation’ refers to managers and professionals in domains such as science, engineering, health, teaching, or clerical office work; ‘manual occupation’ refers to manual labourers, agricultural, services, and sales workers, craft and related trade workers, housewives, and other basic occupations.

#### Potential covariates

Age, sex, and site of residence were collected. For purposes of comparing frequencies of drug use, the Saint-Hyacinthe and Kingston sites were collectively identified as Canadian sites, while Manizales and Natal were identified as Latin American sites.

Depression and the number of chronic illnesses were taken into account, both to measure health needs and as possible confounding variables. Depression was identified using a screening tool for states of emotional functioning, the Center for Epidemiologic Studies Depression Scale (CES-D) [21]. A score <16 indicated the absence of elevated depressive symptomology, while a score ≥16 indicated the presence of elevated symptomology possibly representing depression.

Chronic illnesses taken into account in this study were high blood pressure, diabetes, chronic pulmonary diseases (chronic bronchitis, emphysema, asthma), heart diseases (angina pectoris, heart failure), stroke, and chronic joint damage. For each one, participants were asked whether a health worker had ever told them that they had the illness. Responses were grouped into three categories according to the number of chronic illnesses reported: zero to one, two to three, and four or more.

#### Statistical analyses

The proportions of psychotropic drug users were compared using bivariate analyses based on socioeconomic and demographic factors. We conducted sex-specific analyses to document social differences in psychotropic drug use in men and in women separately. The frequencies of users of anxiolytics, sedatives, and hypnotics, of antidepressants, of analgesics/antiepileptics/antiparkinsonians, and of at least one psychotropic drug were compared through bivariate analysis using chi-square tests. As the use of psychotropic drugs is a generally frequent phenomenon (more frequent than 10 %) and as we were seeking a direct estimation of the prevalence ratio (PR), we adjusted the Poisson regression with a robust variance correction [22]. Given the similarities between the distributions of Kingston and Saint-Hyacinthe on the one hand and those of Natal and Manizales on the other, we pooled the data of the Canadian cities and of the Latin American cities to increase precision of the estimates in multivariate analysis.

The analyses were conducted using version 19 of the Statistical Package for the Social Sciences (SPSS 19). For all tests, the significance threshold was 0.05.

#### Ethical considerations

The IMIAS study was approved by the research ethics committees of the University of Caldas (Colombia), the Universidade Federal do Rio Grande do Norte (Brazil), the Albanian Institute of Public Health (Albania), Queens University (Canada), and the University of Montreal Hospital Research Centre (Canada). Written informed consent was obtained from all subjects before their participation.

## Results

Our survey involved 1,995 participants after screening for dementia. Ten people were excluded: one each in Kingston, Saint-Hyacinthe, and Tirana, two in Manizales, and five in Natal. All participants who reported taking medication were able to show the corresponding drug containers.

Table 1 shows the distribution of psychotropic drug users and non-users according to selected demographic and socioeconomic characteristics as well as mental and physical morbidity. Women used more psychotropic medication than men in three out of the five cities. Older age was related to increased use in Tirana and Manizales, while low income was related to higher use in the Canadian cities. Depression was related to higher use in Kingston, Manizales, and Natal, and comorbidity was related to higher use at all sites except Tirana (*p* = 0.72) and Kingston (*p* = 0.22).

**Table 1:** Description (in %) of the study sample by research site (user and non-user refer to any psychotropic drug use)

	Kingston		Saint-Hyacinthe		Tirana		Manizales		Natal	
	Users	Non-users	Users	Non-users	Users	Non-users	Users	Non-users	Users	Non-users
	(*n* = 108)	(*n* = 290)	(*n* = 123)	(*n* = 278)	(*n* = 118)	(*n* = 276)	(*n* = 97)	(*n* = 303)	(*n* = 38)	(*n* = 364)
Sex										
Male	40.7	49.0	35.0	53.2	47.5	47.8	39.2	52.8	18.4	50.8
Female	59.3	51.0	65.0	46.8	52.5	52.2	60.8	47.2	81.6	49.2
*P-value*	0.175		0.001		1.000		0.020		0.000	
Age										
65–69	45.4	56.5	61.8	64.4	40.7	53.3	43.3	56.8	47.4	54.1
70–74	54.6	43.5	38.2	35.6	59.3	46.7	56.7	43.2	52.6	45.9
*P-value*	0.735		0.653		0.028		0.026		0.495	
Education										
< secondary	01.85	00.00	08.94	06.12	09.32	12.32	78.35	72.28	76.32	78.02
≥ secondary	98.15	100.00	91.06	93.88	90.68	87.68	21.65	27.72	23.68	21.98
*P-value*	0.073		0.297		0.490		0.289		0.838	
Income										
Low/medium	52.7	41.7	78.9	65.8	69.5	68.1	82.5	81.5	68.4	63.5
high	47.2	57.6	17.1	30.9	30.5	31.9	17.5	18.5	31.6	36.5
*P-value*	0.069		0.004		0.814		0.881		0.598	
Occupation										
Non-manual	75.0	76.9	45.5	50.0	39.8	34.8	11.3	16.8	15.8	09.9
Manual	25.0	23.1	54.5	50.0	60.2	65.2	88.7	83.2	84.2	90.1
*P-value*	0.692		0.449		0.361		0.259		0.264	
Depression										
No	85.2	94.1	87.8	91.4	59.3	64.1	67.0	79.9	50.0	83.5
Yes	14.8	05.9	12.2	08.6	40.7	35.9	33.0	20.1	50.0	16.5
*P-value*	0.007		0.276		0.366		0.013		0.000	
Chronic conditions										
Zero or one	36.1	45.9	37.4	51.8	26.3	30.1	40.2	60.4	10.5	42.3
Two or three	51.9	43.8	52.0	38.1	55.1	53.3	49.5	34.7	55.3	46.4
Four and more	12.0	10.3	10.6	10.1	18.6	16.6	10.3	04.9	34.2	11.3
*P-value*	0.217		0.022		0.722		0.002		0.000	
AEP (yes)	40.7		54.5		80.5		74.2		60.5	
ASH (yes)	22.2		34.1		22.0		02.1		26.3	
ADP (yes)	56.5		34.1		03.4		27.8		28.9	

Table 2 presents psychotropic drug use among men and women by site. In Latin America and Canada, women’s frequency of use of AEP and MSN drugs was higher than that of men. Only in Canada did women use ADP drugs more than did men. In Albania, no difference was observed between men’s and women’s use of psychotropic drugs. At all sites, no difference was observed between men’s and women’s use of ASH drugs.

**Table 2:** Prevalence (%) of psychotropic drug use by sex for sites in Canada, Latin America, and Albania

Psychotropic drugs	Sex	Latin America (Natal + Manizales)			Canada (Kingston + Saint-Hyacinthe)			Albania (Tirana)		
		N = 802	(%)	*P*-value	*N* = 799	(%)	*P*-value	*N* = 394	(%)	*P*-value
Anxiolytics, sedatives, and hypnotics (ASH)	Male	390	1.3	0.627	377	6.9	0.186	188	5.3	0.328
	Female	412	1.7		422	9.5		206	7.8	
Antidepressants (ADP)	Male	390	3.8	0.247	377	9.0	0.002	188	1.6	0.272
	Female	412	5.6		422	16.4		206	0.5	
Analgesics/ antiepileptics/antiparkinsonians (AEP)	Male	390	7.2	<0.001	377	10.9	0.020	188	23.9	0.938
	Female	412	16.3		422	16.6		206	24.3	
At least one psychotropic drug (MSN)	Male	390	11.5	< 0.001	377	23.1	0.001	188	29.1	0.947
	Female	412	21.8		422	34.1		206	30.1	

*P*-value were obtained by Chi-square test

At all sites, ASH drug use was not associated with any of the sociodemographic characteristics studied (education, income, occupation) (Table 3). Use of at least one psychotropic drug (MSN) was negatively associated with participant income in Canada. In Latin America only, ADP drug use was negatively associated with education, and participants with non-manual occupations used ADP drugs more than did those in manual occupations. AEP drug use was negatively associated with participant income in Latin America and in Canada. But only in Latin America was the frequency of AEP drug use higher among those with manual professions. In Albania, we did not find any association between use of the various psychotropic drugs and the sociodemographic characteristics studied (Table 3).

**Table 3:** Prevalence (%) of psychotropic drug use according to education, income, and occupation for sites in Canada, Latin America, and Albania

Psychotropic drugs	Socioeconomic variables		Latin America (Natal + Manizales)			Canada (Kingston + Saint-Hyacinthe)			Albania (Tirana)		
			*N* = 802	Percent	*P*-value	*N* = 799	Percent	*P*-value	*N* = 394	Percent	*P*-value
Anxiolytics, sedatives, and hypnotics (ASH)	Education	< Secondary	608	1.8	0.196	30	10.0	0.724	45	6.7	0.984
		Secondary or higher	194	0.5		769	8.2		349	6.6	
	Income	Low/Medium	584	1.0	0.073	458	9.4	0.141	270	5.2	0.095
		High	218	2.8		325	6.5		124	9.7	
	Occupation	Manual	698	1.6	0.630	300	10.3	0.099	251	6.4	0.812
		Non-manual	104	1.0		499	7.0		143	7.0	
Antidepressants (ADP)	Education	< Secondary	608	3.8	0.024	30	23.3	0.082	45	2.2	0.391
		Secondary or higher	194	7.7		769	12.5		349	0.9	
	Income	Low/Medium	584	4.6	0.802	458	14.6	0.147	270	0.7	0.423
		High	218	5.0		325	11.1		124	1.6	
	Occupation	Manual	698	3.7	< 0.001	300	13.3	0.772	251	1.6	0.129
		Non-manual	104	11.5		499	12.6		143	0.0	
Analgesics/ antiepileptics/antiparkinsonians (AEP)	Education	< Secondary	608	13.0	0.075	30	23.3	0.128	45	20.0	0.493
		Secondary or higher	194	8.2		769	13.5		349	24.6	
	Income	Low/Medium	584	13.7	0.008	458	17.5	< 0.001	270	25.2	0.462
		High	218	6.9		325	8.6		124	21.8	
	Occupation	Manual	698	12.9	0.017	300	16.3	0.122	251	22.3	0.268
		Non-manual	104	4.8		499	12.4		143	27.3	
At least one psychotropic drug (MSN)	Education	< Secondary	608	17.3	0.558	30	43.3	0.076	45	24.4	0.392
		Secondary or higher	194	15.5		769	28.3		349	30.7	
	Income	Low/Medium	584	18.2	0.103	458	33.6	< 0.001	270	30.4	0.788
		High	218	13.3		325	22.2		124	29.0	
	Occupation	Manual	698	16.9	0.887	300	31.3	0.242	251	28.3	0.340
		Non-manual	104	16.3		499	27.5		143	32.9	

*P*-value were obtained by Chi-square test

Tables 4, 5, and 6 show the prevalence ratios of psychotropic drug use by socioeconomic indicators, after adjusting for age, sex, depression, and number of chronic illnesses. ASH drug use was not significantly associated with education or with occupation at any of the sites. In Albania, however, persons with low to middle incomes had a significantly lower frequency of ASH drug use than those with high income (PR = 0.43; 95 % CI 0.20–0.94) (Table 4).

**Table 4:** Prevalence ratios of use of anxiolytics, sedatives, and hypnotics (ASH) according to education, income, and occupation for sites in Canada, Latin America, and Albania

Socioeconomic variables		Latin America (Natal + Manizales)			Canada (Kingston + Saint-Hyacinthe)			Albania (Tirana)		
		PR	95 % CI	*P*-value	PR	95 % CI	*P*-value	PR	95 % CI	*P*-value
Education	< Secondary	3.07	0.45–21.20	0.253	0.82	0.26–2.66	0.747	0.84	0.25–2.78	0.774
	Secondary or higher	Ref.			Ref.			Ref.		
	Site 1	0.27	0.06–1.21	0.087	0.58	0.36–0.93	0.024			
	Site 2	Ref.			Ref.					
Income	Low/medium	0.40	0.13–1.22	0.109	1.14	0.67–1.94	0.634	0.43	0.20–0.94	0.034
	High	Ref.			Ref.			Ref.		
	Site 1	0.33	0.07–1.54	0.159	0.62	0.38–1.01	0.055			
	Site 2	Ref.			Ref.					
Occupation	Manual	1.44	0.18–11.22	0.729	1.25	0.75–2.07	0.397	0.87	0.41–1.84	0.709
	Non-manual	Ref.			Ref.			Ref.		
	Site 1	0.27	0.06–1.15	0.077	0.63	0.38–1.04	0.073			
	Site 2	Ref.			Ref.					

Controlled for site, age and sex, depression, and number of chronic illnesses, as well as comparison between grouped sites, if applicable

In Latin America, site 1 = Manizales and site 2 = Natal; In Canada, site 1 = Kingston and site 2 = Saint-Hyacinthe

**Table 5:** Prevalence ratios of antidepressant (ADP) use according to education, income, and occupation for sites in Canada, Latin America, and Albania

Socioeconomic variables		Latin America (Natal + Manizales)			Canada (Kingston + Saint-Hyacinthe)			Albania (Tirana)		
		PR	95 % CI	*P*-value	PR	95 % CI	*P*-value	PR	95 % CI	*P*-value
Education	< Secondary	0.39	0.20-0.73	0.003	2.55	1.23-5.26	0.011	----	----------	---------
	Secondary or higher	Ref.			Ref.					
	Site 1 vs Site 2	2.52	1.30–4.91	0.006	1.61	1.09–2.39	0.017			
Income	Low/Medium	0.53	0.26–1.07	0.078	1.27	0.86–1.89	0.230	----	----------	--------
	High	Ref.			Ref.					
	Site 1 vs Site 2	2.96	1.47–5.99	0.002	1.52	1.04–2.24	0.032			
Occupation	Manual	0.31	0.16–0.59	< 0.001	1.31	0.88–1.92	0.178	----	----------	---------
	Non-manual	Ref.			Ref.					
	Site 1 vs Site 2	2.47	1.26–4.83	0.008	1.59	1.10–2.31	0.014			

Controlled for site, age and sex, depression, and number of chronic illnesses, as well as comparison between grouped sites, if applicable

In Latin America, site 1 = Manizales and site 2 = Natal; In Canada, site 1 = Kingston and site 2 = Saint-Hyacinthe; Number of ADP users in Tirana was very low (n=4)

**Table 6:** Prevalence ratios of analgesics/antiepileptics/antiparkinsonians (AEP) use according to education, income, and occupation for sites in Canada, Latin America, and Albania

Socioeconomic variables		Latin America (Natal + Manizales)			Canada (Kingston + Saint-Hyacinthe)			Albania (Tirana)		
		PR	95 % CI	*P*-value	PR	95 % CI	*P*-value	PR	95 % CI	*P*-value
Education	< Secondary	1.36	0.81–2.29	0.247	1.35	0.62–2.94	0.447	0.78	0.42–1.45	0.440
	Secondary or higher	Ref.			Ref.			Ref.		
	Site 1 vs Site 2	3.53	2.28–5.47	< 0.001	0.66	0.46–0.96	0.028			
Income	Low/Medium	1.13	0.66–1.95	0.651	1.64	1.06–2.54	0.025	1.11	0.75–1.66	0.598
	High	Ref.			Ref.			Ref.		
	Site 1 vs Site 2	3.42	2.20–5.31	< 0.001	0.75	0.52–1.08	0.124			
Occupation	Manual	2.63	1.07–6.44	0.034	1.16	0.81–1.68	0.411	0.82	0.58–1.17	0.278
	Non-manual	Ref.			Ref.			Ref.		
	Site 1 vs Site 2	3.57	2.30–5.54	< 0.001	0.68	0.47–0.98	0.037			

Controlled for site, age and sex, depression, and number of chronic illnesses, as well as comparison between grouped sites, if applicable

In Latin America, site 1 = Manizales and site 2 = Natal; In Canada, site 1 = Kingston and site 2 = Saint-Hyacinthe

After adjustment, ADP drug use was negatively associated with education in Latin America (PR = 0.39; 95 % CI 0.20–0.73), while the reverse was true in Canada (PR = 2.55; 95 % CI 1.23–5.26). Among manual workers in Latin America, ADP drug use was 70 % less than that observed among non-manual workers (PR = 0.31; 95 % CI 0.16–0.59). There was no association between income and ADP drug use (Table 5).

After adjustment, there was no significant association between education and AEP drug use at any sites. An association with income was found only in Canada, where persons with low income showed a prevalence of AEP drug use around 60 % higher than that of those with high income (95 % CI 1.06–2.54). And an association with occupation was seen only at the Latin American sites, where persons with manual occupations had a higher frequency of AEP drug use than did those with non-manual occupations (PR = 2.63; 95 % CI 1.07–6.44) (Table 6).

Among users of AEP drugs, 79.4 % of participants (236 out of 301) were using only analgesics. A low number of participants had used antiepileptics or antiparkinsonians (35 in Canada, 2 in Albania, and 25 in Latin America).

## Discussion

This study shows that there are socioeconomic differences related to the use of psychotropic drugs among women and men elderly persons living in the community and that these associations go in different directions in Canada and outside of Canada. Our results show that income is the most relevant indicator of socioeconomic differences in the use of psychotropic drugs.

In Canada, a negative association was observed between socioeconomic status and psychotropic drug use. Conversely, in Latin America, persons with high income made greater use of psychotropic drugs. In Albania, antidepressants were not used, but the frequency of use of ASH and AEP drugs was almost as high as in Canada. Also the frequency of use of ASH among Albanian elderly persons was higher for those with high income.

### Differences between countries

The frequency of use of psychotropic drugs was much higher in Canada than in the sites outside Canada. These differences between research sites remained after adjusting for age and sex, depression, and chronic illnesses. The differences in use between countries might be explained by the accessibility of psychotropic drugs in each country’s healthcare system and by doctors’ training on the therapeutic effects of these drugs for elderly persons. In Canada and Brazil, a medical prescription is required to obtain ASH and ADP medication. In Colombia and Albania, these medical drugs are readily available over the counter without medical prescription. However, the IMIAS database does not have information on the proportion of medication acquired over the counter.

The low frequency of antidepressant use in Albania might be attributable to historical and social phenomena, common to some ex-Soviet countries [23]. First, there is a strong stigmatization of depression, which was generally considered a sign of weakness during Albania’s communist period [24]. Thus Albanians, and in particular the elderly, avoided drawing attention to any suffering linked to depression and would resort to denial if questioned. Persons with depression preferred to disclose clinical signs related to difficulties with sleeping. The second phenomenon is linked to health professionals (pharmacists, general practitioners), whose competencies in clinical management of mental diseases are limited due to insufficient training and who often consider depression to be a psychosis [25]. Thus, prescribers consider antidepressants to be a very powerful form of treatment compared to anxiolytics, sedatives, and hypnotics (such as diazepam), which are preferred by these professionals. Finally, antidepressants are prohibitively costly in comparison to ASH or AEP [26, 27].

### Differences by sex

The prevalence of use of all types of psychotropic drugs was higher among women. Considering drug use by site however, the difference between sexes was weak to non-existent at Canadian sites and very significant at research sites outside of Canada. This gender difference has been previously observed in Brazil with benzodiazepines use [28]. As the male–female gap remained after controlling for socioeconomic variables, it is likely related to general differences between men and women at the non-Canadian sites. At these sites, medical prescribers may be more likely to prescribe these medications to women [29–35]. Additionally men may be less likely to consult for psychiatric symptoms because they perceived them as a threat to their masculinity [36–38]. This is a topic that would benefit from further international research.

### Differences by depressive symptoms and somatic comorbidity

Finally, as we might have expected, frequency of use of anxiolytics, sedatives, and hypnotics, as well as of antidepressants, was related to higher depressive symptomology. Chronic illness comorbidity, on the other hand, was linked to frequency of use of anxiolytics, sedatives, hypnotics, and analgesics/antiepileptics/antiparkinsonians, but not to that of antidepressants. These results are in agreement with previous reports on anxiolytics (benzodiazepines) use in Brazil [39].

#### Study strengths and limitations

Our study has certain limitations, which need to be kept in mind. The first is that the Kingston study sample over-represents highly educated older adults when compared to the reference population of the 2006 Canadian census data. This could have distorted the observed associations. However, the distribution of physical performance in Kingston (gait speed, balance, and time to stand up from a chair) was very similar to that in Saint-Hyacinthe, the second Canadian city in our study, which was representative in terms of the education, income, and marital status of its reference population. A second limitation is the variation in the meaning of the socioeconomic variable categories. In developed countries such as Canada, manual labour does not have the same implications as it does in middle-income countries. Indeed, the income of elderly persons at the Canadian sites was much higher than that observed at sites in other countries. This is obviously related to the differences in levels of economic development in the countries involved in our study. Standards of education are not the same at the various sites, and education levels of elderly persons are not consistent for the purposes of categorizing the corresponding variable [40, 41]. In our work, each socioeconomic variable was subdivided into two levels to account for the difficulties of comparison linked to the level of economic development of the countries in which study sites were selected. Thus, to help interpret the measures of association, the estimations of associations for the Canadian, Latin American, and Albanian sites were presented by considering the site to be a proxy for the healthcare system, society, and culture of each region. A third limitation concerns the assessment of psychotropic drug use among elderly persons, which was based on use during the two weeks preceding the day of data collection. As such, chronic use and occasional use of psychotropic drugs were considered equivalent, although they are in fact quite different. A fourth limitation is that the IMIAS study did not collect information on elderly persons’ beliefs and attitudes with regard to psychotropic drugs, which could be associated with socioeconomic position (in particular education and occupation) and with drug use. Finally, sample size is the fifth limitation. Our results on the association between level of education and the use of ASH, ADP and AEP have to be considered with caution in the case of older Canadian adults, since only 30 people in Canada were in the lowest education category. The lack of significant results for some types of drugs is due to small numbers. As the numbers in some categories of variables did not permit analysis by individual sites, we had to perform analyses by region while controlling for each site.

Despite these limitations, there are clear strengths to our study. First of all, it is the first study of its kind in the countries outside of Canada. We did not find any published research on social differences in psychotropic drug use among older adults in these countries. Our study is also one of the first to examine social differences in psychotropic drug use while taking into account these three indicators of socioeconomic position. Another strength was our use of a uniform and rigorous data collection methodology across all five sites. The study was thus able to establish the existence of socioeconomic differences associated with psychotropic drug use between elderly men and women living in different economic contexts.

Finally, given the lack of studies on the use of psychotropic drugs among elderly persons in Brazil, Colombia, and Albania, there is a need for research on the impact of such drug use on the health of the elderly. The role of prescribers in the high use of psychotropic drugs among poorer elderly persons in these countries could be a new avenue for research. An increase in self-medication with analgesic drugs may reflect increasing availability and autonomy of community pharmacists or may indicate abuse of painkillers and anxiolytics to increase general well-being. To generalize our findings and answer these questions, our results would have to be validated through further studies, in different settings.

## Conclusion

There are social differences in the use of psychotropic drugs, but they vary according to societies and healthcare systems. Income appears to be the most relevant factor for predicting social differences related to psychotropic drug use in the elderly population. In Canada, greater antidepressant use was associated with a low level of education and low income, and greater analgesic/antiepileptic/antiparkinsonian drug use was associated with low income. In Latin America, a different pattern emerged. Greater antidepressant use was associated with high education level and strong income, while greater analgesic/antiepileptic/antiparkinsonian drug use was associated with manual occupations. In Albania, antidepressants were not used, and anxiolytics, sedatives, and hypnotics were used more frequently by people with high income.
